# Education counteracts the genetic risk of Alzheimer’s disease without an interaction effect

**DOI:** 10.3389/fpubh.2023.1178017

**Published:** 2023-08-17

**Authors:** Xuping Li, Yushi Zhang, Chengcheng Zhang, Ying Zheng, Ruilin Liu, Shuiyuan Xiao

**Affiliations:** ^1^Xiangya School of Public Health, Central South University, Changsha, Hunan, China; ^2^Yale School of Public Health, New Haven, CT, United States; ^3^The Clinical Center for Gene Diagnosis and Therapy, The Second Xiangya Hospital of Central South University, Central South University, Changsha, China

**Keywords:** education, genetic risk, Alzheimer’s disease, dementia, polygenic risk score

## Abstract

**Background:**

Alzheimer’s disease (AD) is a major cause of disability and mortality in older adults. This study aimed to investigate the association of AD with education and genetic factors.

**Methods:**

We conducted a prospective cohort study using data from the UK Biobank. Genetic risk was assessed using a polygenic risk score for AD. The educational level was categorized as either low, intermediate, or high. AD was defined using the International Classification of Diseases and Related Health Problems, 10th revision. Logistic regression models were used to investigate the independent and combined effects of genetic factors and educational levels on the risk of AD.

**Results:**

We included 318,535 participants in this study (age: 56.53 ± 8.09 years; male: 44.81%). Compared with a low genetic risk, a high genetic risk was associated with a significantly greater risk of AD (OR = 7.09, 95% CI: 6.09–8.26). A high educational level was associated with a 30% lower risk of AD compared with a low educational level (OR = 0.70, 95% CI: 0.60–0.81). Combining genetic risk and education categories, individuals with a low genetic risk and high educational level had a more than 90% (OR = 0.09, 95% CI: 0.05–0.16) lower risk of AD compared to those with a high genetic risk and low educational level. There was no significant interaction between genetic risk and educational level regarding AD risk (*p* for interaction = 0.359).

**Conclusion:**

Education counteracts the genetic risk of AD, without an interaction effect. Increasing education to reduce the incidence of AD is of same importance across individuals with different genetic risk.

## 1. Introduction

Dementia is a major cause of disability and death in older adults and imposes a significant economic burden (1–3). The World Health Organization estimated that approximately 50 million people worldwide had dementia in 2019, equating to an economic burden of 1.3 trillion US dollars (4). The global prevalence of dementia has been steadily increasing, and the number of people with dementia has been predicted to reach 130 million by 2050 (5). Alzheimer’s disease (AD) accounts for approximately 70% of all cases of dementia worldwide (6). Given the scale and impact of this disease, reducing its incidence is of paramount importance.

Previous reviews have identified a multitude of factors related to AD, including genetic, demographic, socio-behavioral, and biological factors (7–9). Genetic factors have been recognized as the primary cause of AD in humans (10). A series of twin studies showed that 60–80% of the risk of developing AD is attributable to genetic factors (11). Genome-wide association studies have identified gene polymorphisms associated with the development of AD (12, 13). Single nucleotide polymorphisms have provided quantitative measures of genetic susceptibility, and the polygenic risk score (PRS) has been used to quantify the genetic risk of AD by integrating information from sensitive genetic loci found in genome-wide association studies (14, 15).

A low educational level is recognized as a risk factor for the development of AD (16, 17). A systematic review and meta-analysis of factors associated with AD risk reported odds ratios (ORs) of 2.61 and 1.88 in prevalence and incidence studies, respectively, for low education versus high education (18). The mediating effect of intelligence on educational attainment and AD risk has been demonstrated by a Mendelian randomization study (19). Cognitive reserve refers to the total amount of cognitive resources that individuals are able to mobilize in the face of cognitive challenges. Evidence from a brain structural image analysis showed that education increased the cognitive reserve against AD by increasing regional cortical thickness in healthy controls; this effect also helped AD patients to cope better with the effects of brain atrophy (20).

New strategies for disease prevention have comprised the exploration of gene–environment interactions and the attenuation of genetic risk *via* health-promoting factors (21–23). Previous studies have investigated the interaction between the genetic factors of AD and environmental variables, such as diet, alcohol, smoking, and pollutants (24–27). A study has found education has the same effect on the risk of AD among individuals with APOEε4 gene or without (28). This study aims to explore whether the association between education and the risk of AD was the same at different levels of genetic risk (using PRS) for AD by analyzing data from a large-scale United Kingdom (UK) population cohort. Confirmation of this interaction would help to predict the risk of AD more accurately and inform the development of optimal AD prevention strategies.

## 2. Materials and methods

### 2.1. Study design and participants

This study used data from the UK Biobank, a prospective population-based cohort study. The UK Biobank recruited 502,528 adults (56.53 ± 8.09 years old) from the general population across 22 assessment centers in England, Scotland, and Wales between 2006 and 2010. Participants completed touchscreen and nurse-led questionnaires, underwent physical measurements, and provided biological samples (29). The exposures of interest in this study were genetic risks and educational levels. We included all participants who had complete data on the standard PRS for AD, age at completion of continuous full-time education, and covariates at baseline.

### 2.2. Genetic risk

To assess the cumulative genetic risk of AD, we used the PRS, which was developed based on external genome-wide association summary statistical AD data in the Genetic Epidemiology Research on Adult Health and Aging study (30). An individual-level polygenic score was defined as the sum of the number of risk alleles present at each single nucleotide polymorphism, weighted by the corresponding posterior effect sizes across all available single nucleotide polymorphisms (31). As the genetic risk of AD in a population is approximately normal, most people have an intermediate risk. We categorized the genetic risk for all included individuals into low (<20%), intermediate (20–80%), and high (>80%) risk categories; this classification system has been widely used in other studies (32, 33).

### 2.3. Assessment of education

We used the age at the completion of continuous full-time education to assess the degree of education. This information was collected using touchscreen and nurse-led questionnaires at baseline. Participants were asked the following question: “At what age did you complete your continuous full-time education?.” The participants were categorized as having either a low (the age at the completion of continuous full-time education: <16 years), intermediate (the age at the completion of continuous full-time education: 16–18 years), or high (the age at the completion of continuous full-time education: >18 years) educational level.

### 2.4. Ascertainment of AD

AD was defined according to the International Classification of Diseases and Related Health Problems, 10th revision, which is the primary classification system used by the UK Biobank. We used code G30 from the “first occurrence” data field generated by the UK Biobank. This code was ascertained by combining primary care center, hospital inpatient, death register, and self-reported data. The date of diagnosis was set as the earliest date on which the AD code was recorded, regardless of the source used.

### 2.5. Covariates

Covariates were selected based on previous research and baseline availability (34–36). Demographic variables, including sex (male and female) and age at baseline (categorized as <45, 45–49, 50–54, 55–59, 60–64, 65–69, and ≥ 70 years), as well as health behaviors such as smoking status (current or non-current), alcohol intake (<3 times per week or ≥ 3 times per week), and moderate physical activity (0, 1–2, 3–5, and 6–7 times per week) were obtained *via* touchscreen and nurse-led questionnaires at baseline. These measures of health behaviors have been widely used in other studies (32, 36). Biological factors including body mass index (BMI) (<25, 25–30, and > 30), diabetes, and hypertension were also assessed, as previous studies have reported that obesity, diabetes, and hypertension are risk factors for AD (36). BMI was calculated by dividing self-reported weight (kg) by height (m^2^) at baseline. Diabetes and hypertension were determined via medical records.

### 2.6. Statistical analysis

Logistic regression was used to investigate the association of AD with genetic risk, education, and combined genetic risk and education categories. ORs was used to show the relative strength of the risk of AD among different populations after adjusting covariates. To explore the influence of genetic risk and education on AD, these two variables were mutually adjusted. We examined the effects of education on AD development in populations with varying levels of genetic risk. To account for the potential impact of covariates on the association of AD with genetic risk and education, model 1 was adjusted for sex and age at baseline; model 2 was additionally adjusted for smoking status, alcohol intake, and physical activity; and model 3 was further adjusted for BMI, diabetes, and hypertension. Combined genetic risk and education categories were included as variables in the analysis. An interaction term was included in the regression model to test for statistical interactions between genetic risk and education in relation to AD. The effect of education on AD was also analyzed by stratifying for genetic risk. Bonferroni correction was made to reduce the probability of false positives.

### 2.7. Sensitivity analysis

We conducted two types of sensitivity analyses to evaluate the robustness of our findings. The first analysis involved re-categorizing PRS into low (<30%), intermediate (30–70%), and high (>70%) risk categories for all included individuals. The second sensitivity analysis explored potential differences in the risk of AD between males and females.

The significance level was set at α = 0.05, and two-sided *p*-values less than 0.05 were considered statistically significant. All analyses were performed using SPSS.22 statistical software and R.4.1.2.

## 3. Results

### 3.1. Participants in the study

A total of 502,386 individuals were assessed at the baseline. After excluding participants without data pertaining to genetic factors (*n* = 16,237), educational level (*n* = 165,186), BMI (*n* = 3,105), health behaviors (*n* = 12,898), or diabetes and hypertension status (*n* = 13,823), a total of 318,535 participants were finally included in the study. During the study, 2,483 persons were diagnosed with AD. The participants’ characteristics are shown in Table 1.

**Table 1:** Characteristics of participants included.

Characteristics	All	AD	Non-AD	*p* value
Age at baseline				<0.001
<45 years	28,070 (8.81)	6 (0.02)	28,064 (99.98)	
45 ~ 49 years	38,057 (11.95)	18 (0.05)	38,039 (99.95)	
50 ~ 54 years	44,984 (14.12)	50 (0.11)	44,934 (99.89)	
55 ~ 59 years	55,317 (17.37)	158 (0.29)	55,159 (99.71)	
60 ~ 64 years	82,443 (25.88)	699 (0.85)	81,744 (99.15)	
65 ~ 69 years	67,900 (21.32)	1,479 (2.18)	66,421 (97.82)	
≥70 years	1764 (0.55)	73 (5.27)	1,671 (94.73)	
Sex				0.087
Male	142,744(44.81)	1,155 (0.81)	141,589 (99.19)	
Female	175,791 (55.19)	1,328 (0.76)	174,463 (99.24)	
Genetic risk				<0.001
Low	63,708 (20.00)	191 (0.30)	63,517 (99.70)	
Intermediate	191,120 (60.00)	1,004 (0.53)	190,116 (99.47)	
High	63,707 (20.00)	1,288 (2.02)	62,419 (97.98)	
Education				<0.001
Low	99,375 (31.20)	1,290 (1.30)	98,085 (98.70)	
Intermediate	177,484 (55.72)	980 (0.55)	176,504 (99.45)	
High	41,676 (13.08)	213 (0.51)	41,463 (99.49)	
Smoking status				
current	37,956 (11.92)	227 (0.60)	37,729 (99.40)	
non-current	280,579 (88.08)	2,256 (0.80)	278,323 (99.20)	
Alcohol intake				<0.001
< 3 times/week	251,197 (78.86)	1763 (0.70)	249,434 (99.30)	
≥3 times/week	67,338 (21.14)	720(1.07)	66,618 (98.93)	
Moderate physical activity				<0.001
0 time/week	43,271 (13.58)	317 (0.73)	42,954 (99.27)	
1 ~ 2 times/week	66,617 (20.91)	433 (0.65)	66,184 (99.35)	
3 ~ 5 times/week	128,242 (40.26)	949 (0.74)	127,293 (99.26)	
6 ~ 7 times/week	80,405 (25.24)	784 (0.98)	79,621 (99.02)	
BMI				0.530
<25	94,412 (29.64)	754 (0.80)	94,412 (99.2)	
25 ~ 30	138,032 (43.33)	1,081 (0.78)	138,032 (99.22)	
>30	86,091 (27.03)	648 (0.75)	86,091 (99.25)	
Diabetes				<0.001
Yes	17,206 (5.40)	290 (1.71)	16,916 (98.29)	
No	301,329 (94.60)	2,193 (0.73)	299,136 (99.27)	
Hypertension				<0.001
Yes	86,500 (27.16)	895 (1.03)	85,605 (98.97)	
No	232,035 (72.84)	1,588 (0.68)	230,447 (99.32)	

**Figure 1:**
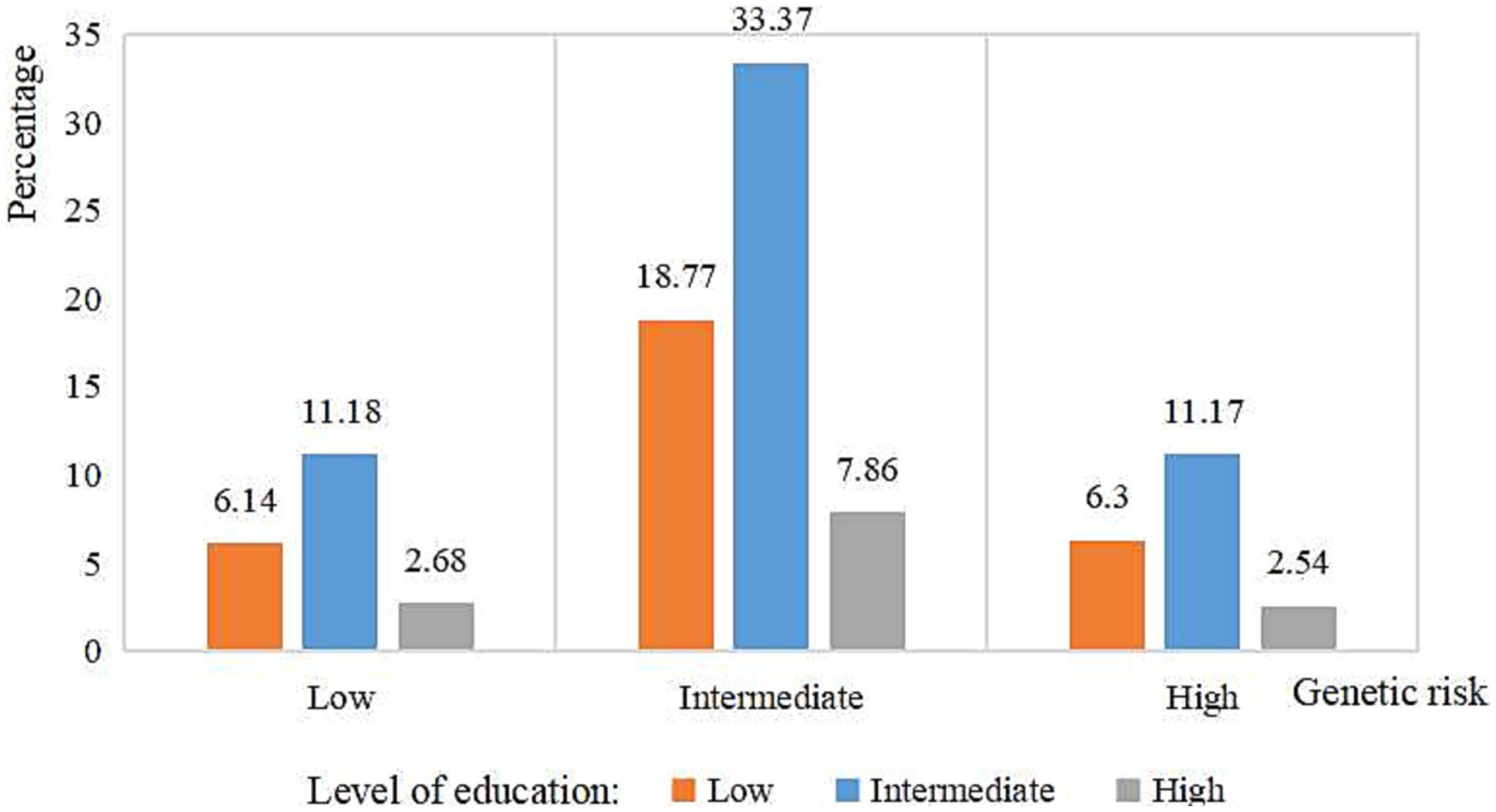
Percentage of level of education according to genetic risk categories risk of AD.

Figure 1 shows the percentages of participants with different levels of genetic risk and education: 31.2, 55.72, and 13.08% had low, intermediate, and high educational levels, respectively. Approximately one-third of participants had an intermediate genetic risk and low educational level, while 2.54% had a low genetic risk and high educational level. Across all levels of genetic risk, the educational level was most frequently classified as intermediate and least frequently classified as high.

### 3.2. The impact of genetic risk on AD

Table 2 presents the proportions and ORs for AD across different levels of genetic risk and education. The lifetime prevalence rates of AD were 0.30, 0.53, and 2.02% at low, intermediate, and high levels of genetic risk, respectively. In model 1 (which was adjusted for sex, age, and education), the ORs of AD were 1.76 (95% CI: 1.51–2.06) and 7.08 (95% confidence interval [CI]: 6.07–8.25) for intermediate and high genetic risk categories, respectively, versus low genetic risk. These results remained unchanged following additional adjustments for behavioral factors in model 2 and for both behavioral and biological factors in model 3. Similar results were observed in the sensitivity analyses for males (Supplementary Table S1), females (Supplementary Table S2), and different categories of genetic risk (Supplementary Table S3). These findings indicated that the genetic risk for AD was statistically independent of other factors.

**Table 2:** Risk of AD according to genetic risk and education categories.

	AD proportion	Model 1	Model 2	Model 3
*Genetic risk*				
Low	0.30% (191/63708)	1 (Ref)	1 (Ref)	1 (Ref)
Intermediate	0.53% (1,004/191120)	1.76 (1.51, 2.06)	1.77 (1.51, 2.06)	1.76 (1.51, 2.06)
High	2.02% (1,288/63707)	7.08 (6.07, 8.25)	7.10 (6.09, 8.27)	7.09 (6.09, 8.26)
*Education*				
Low	1.30% (1,290/238504)	1 (Ref)	1 (Ref)	1 (Ref)
Intermediate	0.55% (980/68019)	0.78 (0.71, 0.85)	0.80 (0.73, 0.87)	0.79 (0.72, 0.86)
High	0.51% (213/12012)	0.70 (0.60, 0.81)	0.71 (0.61, 0.83)	0.70 (0.60, 0.81)

### 3.3. The impact of education on AD

The proportion and risk of AD decreased monotonically across the education categories. As shown in Table 2, a higher educational level was associated with a lower proportion and risk of AD. In model 1 (which was adjusted for sex, age, and genetic risk), ORs were 0.78 and 0.70 for the intermediate and high education groups, respectively, versus the low education group. Similar results were observed following additional adjustments for behavioral factors in model 2 and both behavioral and biological factors in model 3. These results were also essentially unchanged following sensitivity analyses for males (Supplementary Table S1), females (Supplementary Table S2), and different categories of genetic risk (Supplementary Table S3), thus indicating that education was independently associated with AD.

### 3.4. The combined impact of genetic risk and education on AD

**Figure 2:**
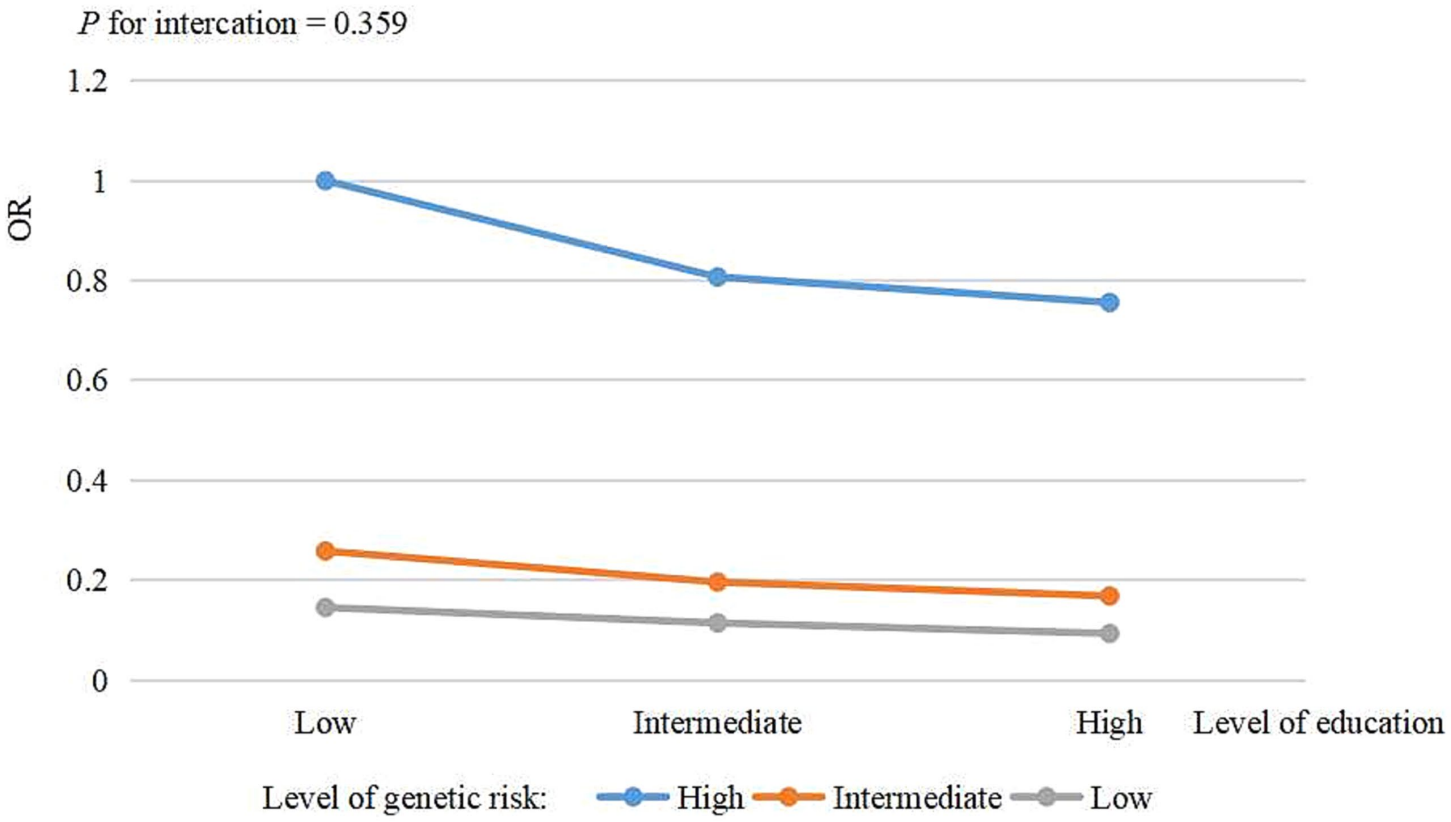
The risk of combined genetic risk and education for AD.

Analyses of combined genetic risk and education categories revealed an overall monotonic association between lower genetic risk and higher education (Figure 2). Participants with a low genetic risk and high educational level had a lower risk of AD compared to those with a high genetic risk and low educational level (OR = 0.09, 95% CI: 0.06–0.15), after adjustment for all covariates. No significant interaction was observed between genetic risk and education in relation to AD (*p* for interaction = 0.359), indicating that the association between AD and education did not vary substantially across different levels of genetic risk. The details of the combined effects of genetic risk and education are shown in Supplementary Table S4. The sensitivity analyses (male: Supplementary Figure S1 and Supplementary Table S5; female: Supplementary Figure S2 and Supplementary Table S6; different categories of genetic risk: Supplementary Table S7 and Supplementary Figure S3) yielded similar results.

### 3.5. The impact of education on AD across different levels of genetic risk

The effect of education on AD following stratification by genetic risk and adjustment for all covariates is shown in Table 3. Compared to a low educational level, intermediate and high educational levels were associated with 21% (OR = 0.79, 95% CI: 0.58–1.07) and 35% (OR = 0.65, 95% CI: 0.38–1.08) lower risks for AD, respectively, among participants with a low genetic risk. Among participants with an intermediate genetic risk, an intermediate educational level was associated with a risk reduction of 24% (OR = 0.76, 95% CI: 0.66–0.87), while a high educational level was associated with a risk reduction of 34% (OR = 0.66, 95% CI: 0.51–0.82). Among participants with a high genetic risk, intermediate and high educational levels were associated with 20% (OR = 0.80, 95% CI: 0.72–0.91) and 25% (OR = 0.75, 95% CI: 0.61–0.92) reductions in risk for AD, respectively. These results were not significantly altered in the sensitivity analyses for males (Supplementary Figure S4), females (Supplementary Figure S5), and the different categories of genetic risk (Supplementary Figure S6).

**Table 3:** The effect of education on AD stratified by genetic risk.

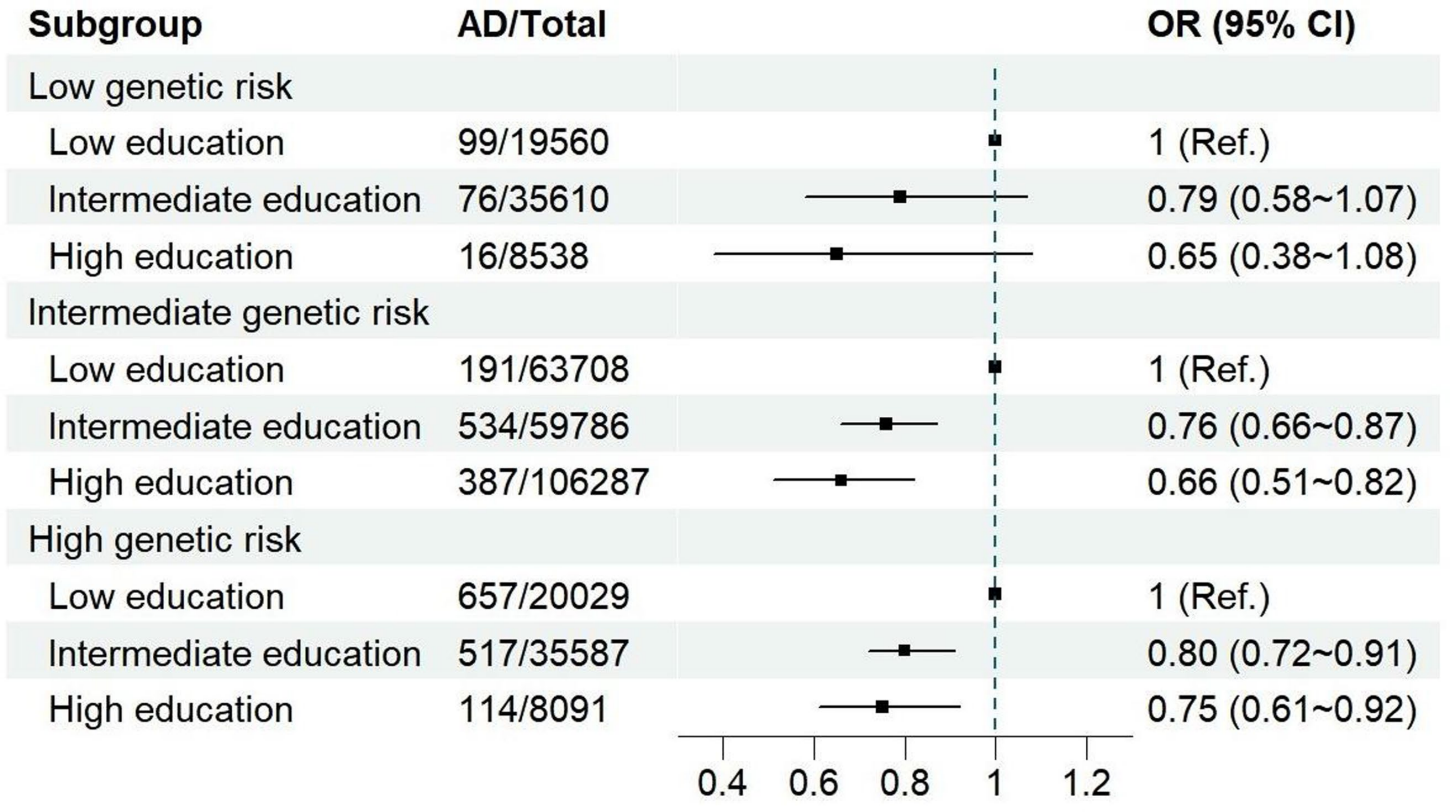

## 4. Discussion

The 1970s and 1980s saw major changes in the British educational system. Universal access to general and vocational education increased significantly, with a 50% high school enrollment rate (37). Most of the participants in this study completed their education during this period. Individuals in the UK typically complete high school at 16–18 years of age (38). They then have the option of pursuing higher education or entering the workforce. In our study, the distribution of educational levels among the participants reflected the overall trend in the UK at that time.

In this prospective, population-based, large-scale study, we found that genetic risk and education were associated with the risk of AD. This finding is consistent with previous studies (16, 17, 28). After adjusting for education, individuals with a high genetic risk were six times more likely to develop AD than those with a low genetic risk. This suggests that identifying individuals at high genetic risk could facilitate a more precise targeting of individuals for AD prevention. After adjusting for genetic risk, the risk of AD decreased by 30% among participants with a high educational level compared to those with a low educational level. The risk decreased by 21% even among participants with an intermediate level of education. No significant interaction was observed between genetic risk and educational level. A beneficial effect of higher education on AD was found across all levels of genetic risk. This suggests that the incidence of AD could be reduced at a population-wide level through higher education, which was consistent with the research results that used the presence of the APOEε4 gene as a genetic risk grouping (28).

To the best of our knowledge, this is the first study to investigate the association between AD and combinations of different levels of education and genetic risk. Previous studies have shown that education and genetic risk are associated with other diseases, such as obesity and diabetes (39, 40). By analyzing different combinations of genetic risk and education categories, it was found that the risk of AD was decreased by more than 90% in people with a low genetic risk and high educational level, relative to those with a high genetic risk and low educational level. Combining genetic risk and education may facilitate a more precise prediction of AD. Therefore, the adoption of other measures, such as healthy lifestyles, for reducing the risk of AD is particularly important for individuals with a high genetic risk and low educational level (32).

The findings of our study could be explained previous studies (41–45). Primary education plays a crucial role in equipping individuals with fundamental hygiene knowledge and fostering a healthy lifestyle (41). Secondary education, on the other hand, enhances individuals’ comprehension abilities and significantly improves health literacy (42). Furthermore, higher education empowers individuals to access more resources for personal development, thereby further augmenting their capacity to uphold good health (19). Some researchers believe that dementia occurs due to a decline in the cognitive reserve below a certain threshold (43). A higher level of education provides individuals with an increased cognitive reserve, thereby lowering the risk of dementia. Other studies have also suggested that the association between educational attainment and the reduced risk of AD is driven by intelligence. Individuals with higher education develop more cognitive reserve, so they are able to mobilize more resource to prevent AD (19, 45). In addition, educational attainment shapes reactions to genetic risk for AD (43). Individuals with a higher educational level tend to exhibit more health-promoting behaviors, while they have greater material, psychological, and social resources for maintain good health (46).

### 4.1. Limitations

Although this study was based on a large-scale survey, several limitations are acknowledged. First, education was evaluated based on the age at which participants completed continuous full-time education, which may not reflect the exact amount of time spent on education. However, it is likely to reflect a significant level of educational attainment (38). Due to a lack of data on part-time education, this study did not consider the impact of this type of education on AD occurrence. Second, the method used to identify AD cases may have been inadequate. Nonetheless, the use of primary care, hospital inpatient, death register, and self-reported data included more than 82.5% of the relevant records (47). Third, some researchers hold the view that individuals with high education still get AD, however, the symptoms of AD are often staved off for a longer period of time (48). This study did not consider the differences in this delayed effect among different genetic risk groups. Fourth, the relationship between education and AD risk is complicated. Mediating variables of this relationship were not explored in this study. Fifth, this study was restricted to individuals in the UK aged 37–73 years at baseline. Therefore, caution should be exercised when generalizing the findings in this study to other populations.

### 4.2. Implications

The results of this study suggest that higher education is associated with a decreased risk of AD, regardless of genetic risk. Compared to individuals with lower levels of education, even those who have not received higher education but completed secondary education (approximately at ages 16–18) exhibit a significant reduced risk of developing AD. Furthermore, this effect is consistent across different levels of genetic risk. These findings have important implications for population-wide public health practices aimed at preventing AD. Fostering a conducive educational environment and offering abundant educational opportunities (such as free secondary education) is crucial for governments and societies. Additionally, individuals actively pursue higher education also play a significant role in the prevention of AD within the population (49). This study explored the correlation between AD and educational levels, which were based on the age at which continuous full-time education was completed. Future studies should also consider the influence of part-time education on the risk of developing AD. The effect of education on the risk of AD in different genetic risk groups should also be explored from the perspective of higher education delaying the onset of AD.

### 4.3. Conclusion

The results of this study indicate that genetic risk and education are independently associated with the risk of AD. Higher educational attainment may decrease the risk of AD, regardless of an individual’s genetic risk. Hence, it is equally crucial for individuals with varying genetic risk to prioritize augmenting educational attainment in order to reduce the risk of AD. Governments and societies should foster a conducive educational environment, while individuals should actively seize educational resources to maximize their impact.

## Data availability statement

The raw data supporting the conclusions of this article will be made available by the authors, without undue reservation.

## Ethics statement

Ethical approval was not required for the study involving humans in accordance with the local legislation and institutional requirements. Written informed consent to participate in this study was not required from the participants or the participants’ legal guardians/next of kin in accordance with the national legislation and the institutional requirements.

## Author contributions

XL collected the data, processed statistical data, and drafted the manuscript. YuZ, CZ, YiZ, and RL contributed to processed statistical data, and drafted the manuscript. SX contributed to the study design, manuscript edits, and supervised the project. All co-authors read and approved the final manuscript.

## Funding

This research was funded by the Ministry of Science and Technology of China (Grant No. 2016YFC0900802).

## Conflict of interest

The authors declare that the research was conducted in the absence of any commercial or financial relationships that could be construed as a potential conflict of interest.

## Publisher’s note

All claims expressed in this article are solely those of the authors and do not necessarily represent those of their affiliated organizations, or those of the publisher, the editors and the reviewers. Any product that may be evaluated in this article, or claim that may be made by its manufacturer, is not guaranteed or endorsed by the publisher.

## Abbreviations

AD: Alzheimer’s disease
BMI: body mass index
CI: confidence interval
OR: odds ratio
PRS: polygenic risk score
UK: United Kingdom
