# Supplementary material for: Education counteracts the genetic risk of Alzheimer’s disease without an interaction effect
Source: Front Public Health. 2023 Aug 17;11:1178017. doi: 10.3389/fpubh.2023.1178017 (PMC10471486; doi:10.3389/fpubh.2023.1178017)
Supplement: Supplementary file 1 [file Data_Sheet_1.pdf]

Table S1. Risk of AD according to genetic risk and education categories among male

	**AD proportion**	**Model 1**	**Model 2**	**Model 3**
Genetic risk				
Low	95/28317	1 (Ref)	1 (Ref)	1 (Ref)
Intermediate	491/85815	1.73 (1.34, 2.16)	1.74 (1.39, 2.16)	1.73 (1.39, 2.16)
High	569/28612	6.18 (4.96, 7.69)	6.20 (4.99, 7.20)	6.20 (4.98, 7.12)
Education				
Low	631/46522	1 (Ref)	1 (Ref)	1 (Ref)
Intermediate	417/75716	0.76 (0.67, 0.87)	0.78 (0.68, 0.88)	0.77 (0.68, 0.88)
High	107/20506	0.70 (0.56, 0.86)	0.70 (0.57, 0.86)	0.69 (0.56, 0.85)

Table S2. Risk of AD according to genetic risk and education categories among female

	AD proportion	Model 1	Model 2	Model 3
Genetic risk				
Low	96/35391	1 (Ref)	1 (Ref)	1 (Ref)
Intermediate	513/105305	1.80 (1.45, 2.24)	1.80 (1.45, 2.25)	1.80(1.50, 2.25)
High	719/35095	7.94 (6.41, 9.83)	7.96 (6.42, 9.86)	7.98 (6.44, 9.89)
Education				
Low	659/52853	1 (Ref)	1 (Ref)	1 (Ref)
Intermediate	563/101768	0.78 (0.70, 0.88)	0.80 (0.72, 0.91)	0.80 (0.71, 0.90)
High	106/21170	0.70 (0.57, 0.86)	0.72 (0.58, 0.88)	0.70 (0.57, 0.87)

Table S3. Risk of AD according to genetic risk and education categories using different categories of genetic risk

	AD proportion	Model 1	Model 2	Model 3
Genetic risk				
Low	307/95561	1 (Ref)	1 (Ref)	1 (Ref)
Intermediate	633/127413	1.56 (1.36, 1.78)	1.56 (1.36, 1.79)	1.56 (1.36, 1.78)
High	1543/95561	5.21 (4.61, 5.89)	5.22 (4.61, 5.90)	5.22 (4.61, 5.91)
Education				
Low	2084/238504	1 (Ref)	1 (Ref)	1 (Ref)
Intermediate	328/68019	0.78 (0.71, 0.85)	0.80 (0.73, 0.87)	0.80 (0.71, 0.90)
High	71/12012	0.70 (0.60, 0.81)	0.71 (0.61, 0.82)	0.70 (0.57, 0.87)

Table S4. The risk of combined genetic risk and education for AD

Genetic risk * Education	OR (95% CI)
High * Low	1
High * Intermediate	0.81 (0.72, 0.91)
High * High	0.76 (0.61, 0.91)
Intermediate * Low	0.26 (0.23, 0.29)
Intermediate * Intermediate	0.20 (0.17, 0.22)
Intermediate * High	0.17 (0.13, 0.21)
Low * Low	0.15 (0.12, 0.18)
Low * Intermediate	0.11 (0.09, 0.14)
Low * High	0.09 (0.06, 0.15)

Table S5. The risk of combined genetic risk and education for AD among male

Genetic risk * Education	OR (95% CI)
High * Low	1
High * Intermediate	0.78 (0.65, 0.93)
High * High	0.71 (0.53, 0.96)
Intermediate * Low	0.28 (0.24, 0.33)
Intermediate * Intermediate	0.21 (0.18, 0.26)
Intermediate * High	0.20 (0.14, 0.27)
Low * Low	0.16 (0.12, 0.22)
Low * Intermediate	0.13 (0.09, 0.18)
Low * High	0.10 (0.05, 0.19)

Table S6. The risk of combined genetic risk and education for AD among female

Genetic risk * Education	OR (95% CI)
High * Low	1
High * Intermediate	0.82 (0.70, 0.97)
High * High	0.78 (0.59, 1.03)
Intermediate * Low	0.24 (0.20, 0.28)
Intermediate * Intermediate	0.18 (0.15, 0.22)
Intermediate * High	0.14 (0.10, 0.20)
Low * Low	0.13 (0.10, 0.17)
Low * Intermediate	0.10 (0.07, 0.14)
Low * High	0.09 (0.05, 0.18)

Table S7. The risk of combined genetic risk and education for AD using different categories of genetic risk

Genetic risk * Education	OR (95% CI)
High * Low	1
High * Intermediate	0.80 (0.72, 0.89)
High * High	0.78 (0.65, 0.94)
Intermediate * Low	0.31 (0.27, 0.35)
Intermediate * Intermediate	0.24 (0.21, 0.28)
Intermediate * High	0.16 (0.12, 0.22)
Low * Low	0.20 (0.17, 0.24)
Low * Intermediate	0.15 (0.12, 0.18)
Low * High	0.14 (0.09, 0.20)

Table S8. The effect of education on AD stratified by genetic risk among male

Subgroup	AD/Total	OR (95% CI)
Low genetic risk		
Low education	48/10506	1 (Ref.)
Intermediate education	40/20568	0.83 (0.54~1.27)
High education	8/4317	0.74 (0.35~1.59)
Intermediate genetic risk		
Low education	263/31735	1 (Ref.)
Intermediate education	214/60894	0.75 (0.62~0.90)
High education	36/12676	0.59 (0.41~0.83)
High genetic risk		
Low education	348/10612	1 (Ref.)
Intermediate education	309/20306	0.85 (0.71~0.98)
High education	62/4177	0.79 (0.60~1.05)

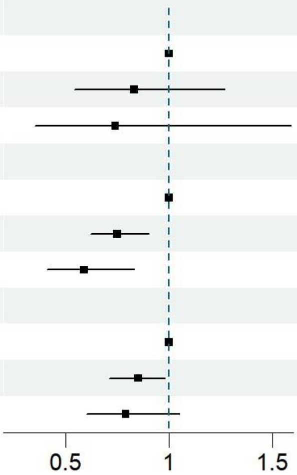

Table S9. The effect of education on AD stratified by genetic risk among female

Subgroup	AD/Total	OR (95% CI)
Low genetic risk		
Low education	51/9054	1 (Ref.)
Intermediate education	36/15042	0.74 (0.48~1.15)
High education	8/4221	0.56 (0.26~1.18)
Intermediate genetic risk		
Low education	271/28051	1 (Ref.)
Intermediate education	173/45393	0.77 (0.63~0.94)
High education	47/12371	0.70 (0.51~0.96)
High genetic risk		
Low education	309/9417	1 (Ref.)
Intermediate education	208/15281	0.77 (0.64~0.93)
High education	52/3914	0.71 (0.53~0.96)

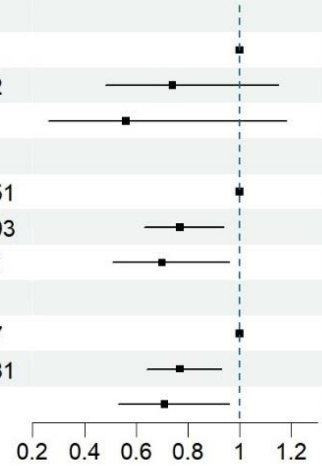

Table S10. The effect of education on AD stratified by genetic risk using different categories of genetic risk

Subgroup	AD/Total	OR (95% CI)
Low genetic risk		
Low education	163/29500	1 (Ref.)
Intermediate education	117/53384	0.75 (0.59~0.96)
High education	27/12677	0.68 (0.45~1.03)
Intermediate genetic risk		
Low education	336/39834	1 (Ref.)
Intermediate education	245/70737	0.79 (0.67~0.93)
High education	43/16842	0.52 (0.38~0.72)
High genetic risk		
Low education	791/30041	1 (Ref.)
Intermediate education	609/53363	0.80 (0.71~0.89)
High education	143/12157	0.79 (0.65~0.94)

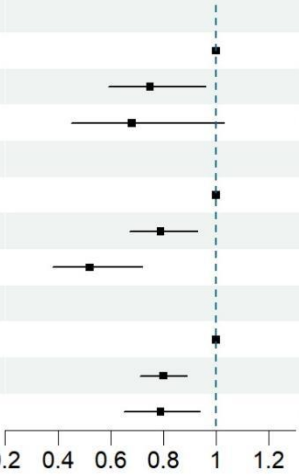

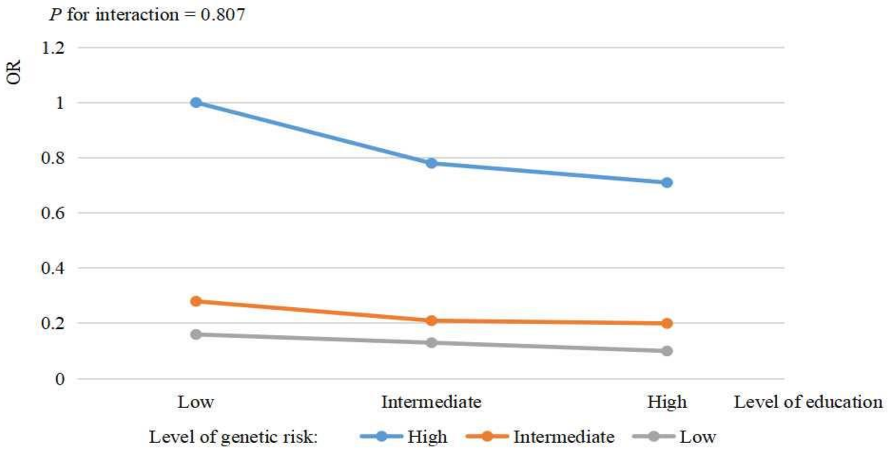

Figure S1. The risk of combined genetic risk and education for AD among male

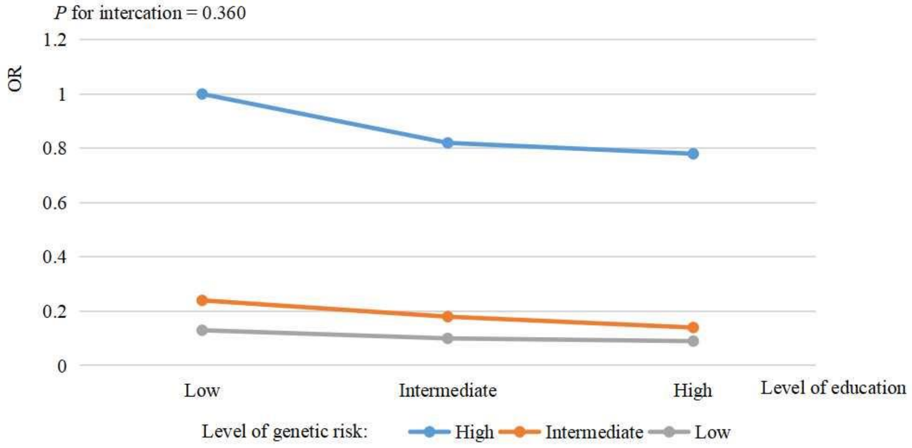

Figure S2. The risk of combined genetic risk and education for AD among female
